# Targeting ion channels with ultra-large library screening for hit discovery

**DOI:** 10.3389/fnmol.2023.1336004

**Published:** 2024-01-05

**Authors:** Kortney Melancon, Palina Pliushcheuskaya, Jens Meiler, Georg Künze

**Affiliations:** ^1^Department of Chemistry, Vanderbilt University, Nashville, TN, United States; ^2^Center for Structural Biology, Vanderbilt University, Nashville, TN, United States; ^3^Medical Faculty, Institute for Drug Discovery, Leipzig University, Leipzig, Germany; ^4^Center for Scalable Data Analytics and Artificial Intelligence, Leipzig University, Leipzig, Germany; ^5^Interdisciplinary Center for Bioinformatics, Leipzig University, Leipzig, Germany

**Keywords:** virtual screening, ion channels, drug design, deep learning, chemical library

## Abstract

Ion channels play a crucial role in a variety of physiological and pathological processes, making them attractive targets for drug development in diseases such as diabetes, epilepsy, hypertension, cancer, and chronic pain. Despite the importance of ion channels in drug discovery, the vastness of chemical space and the complexity of ion channels pose significant challenges for identifying drug candidates. The use of *in silico* methods in drug discovery has dramatically reduced the time and cost of drug development and has the potential to revolutionize the field of medicine. Recent advances in computer hardware and software have enabled the screening of ultra-large compound libraries. Integration of different methods at various scales and dimensions is becoming an inevitable trend in drug development. In this review, we provide an overview of current state-of-the-art computational chemistry methodologies for ultra-large compound library screening and their application to ion channel drug discovery research. We discuss the advantages and limitations of various *in silico* techniques, including virtual screening, molecular mechanics/dynamics simulations, and machine learning-based approaches. We also highlight several successful applications of computational chemistry methodologies in ion channel drug discovery and provide insights into future directions and challenges in this field.

## Introduction

Ion channels are widely expressed in living cells and play critical roles in the generation of the cell membrane potential and in additional diverse cellular functions, such as signal transduction, neurotransmitter release, muscle contraction, hormone secretion, cell volume regulation, growth, mobility, and apoptosis. Dysfunction of ion channels due to mutations in ion channel genes are associated with numerous diseases collectively known as channelopathies, which include cardiac arrhythmias, ataxias, migraine headaches, muscle paralysis, epilepsy, deafness and cancer. More than 60 channelopathies have been identified in human diseases, and clinical sequencing results often discover novel mutations in ion channel genes (Cannon, 2007; Kim, 2014). Because of the high pathophysiological importance of ion channels and their involvement in several human diseases, they are the target of diverse drugs, from antiepileptics to analgesics. Ion channels are the second largest group of drug targets with approximately 130 drugs on the market that act on ion channels. Examples include voltage-gated sodium channel blockers for the treatment of arrhythmia and local anesthesia, calcium channel blockers for the treatment of angina and hypertension, and ATP-sensitive potassium channel blockers for the therapy of type II diabetes (Ford et al., 2016; Imbrici et al., 2016; Santos et al., 2016; Li et al., 2017; Wolkenberg et al., 2017; Hutchings et al., 2019; Chen et al., 2023). Despite this large number of existing drugs, ion channels remain relatively underexploited for therapeutic interventions. It is notable that the major chemical classes of ion channel modulators were identified through serendipity and have been in clinical use for many years, pre-dating major milestones in ion channel research such as the development of patch clamp physiology, molecular cloning of ion channels and their structure determination by cryogenic electron microscopy (cryo-EM).

The rapid growth in structural information of ion channels has fueled the use of computer-assisted drug discovery approaches. At the time of writing (as of October 2023), approximately 100 unique human ion channel protein structures have been deposited in the Protein Data Bank (PDB), ~81% of these structures were determined by cryo-EM (Lau et al., 2018; Rao et al., 2019; Chen et al., 2023; mpstruc database, n.d.). In recent decades, the increasing numbers of high-quality ion channel structures, along with advances in computer-assisted drug discovery, have led to a number of successful virtual screening (VS) campaigns (Kang, 2001; Urbahns et al., 2003; Kenyon et al., 2006; Liu et al., 2007; Etkins, 2018; Llanos et al., 2022; Pasqualetto et al., 2023). Among ion channels, L-type calcium channels and hERG channels have received the most extensive research attention to date (Ekins et al., 2007). In contrast, there are very few examples of successful applications for more ion-selective channels (K, Na) and less selective channels such as nicotinic acetylcholine receptors (nAChR) or acid-sensing ion channels (ASICs).

Most recently, the utility of VS for lead discovery has also been boosted by the expansion of accessible chemical space through make-on-demand compound libraries like Enamine REAL Space library (Grygorenko et al., 2020; Enamine, n.d.). Since 2016, these libraries have witnessed a remarkable expansion, scaling up the availability of molecules from 11 million to an astonishing 38 billion, and there is still potential for further growth (Lyu et al., 2023). While such libraries cannot be empirically screened, molecules within them can be computational prioritized for synthesis and testing using VS and machine learning approaches. Combining *in silico* approaches with conventional high-throughput screening techniques greatly enhances ion channel drug discovery. Methods of computer-aided drug discovery (CADD) can significantly speed up screening and can drastically improve hit rates. Molecular docking is routinely used to process virtual libraries containing millions of molecules against a variety of drug targets with known structures.

Recent strides in automated synthesis and the proliferation of available chemicals present significant opportunities for VS methods overall, and especially for docking. However, they also introduce entirely new challenges to contend with (Gentile et al., 2020). The widely used ZINC library has grown from 7,000 entries in 2005 to over 1.3 billion constituent molecules in 2020, a remarkable 1,000-fold increase (Irwin and Shoichet, 2005; Irwin et al., 2020; ZINC Database, n.d.). In the past two years alone, the Enamine REAL database has grown from 11 billion molecules to 38 billion make-on-demand molecules (Grygorenko et al., 2020). Recently published works seem to advocate for expanding VS to ultra-large chemical libraries. In a recent groundbreaking study, Lyu et al. conducted docking experiments with 170 million on-demand molecular structures (Lyu et al., 2019). Their findings demonstrated that VS of such extensive databases not only enables the discovery of highly potent inhibitors but also uncovers novel chemical classes that are typically absent from routinely screened, readily available chemical libraries (Lyu et al., 2019). Other docking studies involving large collections of molecules led to similar conclusions (Gorgulla et al., 2020; Stein et al., 2020).

Progress in high-throughput docking programs, computational resources, and the accessibility of more and more ion channel structures, promote a paradigm shift in drug discovery research toward faster *in silico* lead compound generation. Despite these advancements, it's worth noting that the chemical space remains so vast that it often remains beyond practical reach. A common approach to mitigate these challenges is to filter these large chemical collections to manageable subsets based on parameters set forth by Lipinski and others (Lipinski et al., 2001; Lipinski, 2004). While this approach can effectively reduce an ultra-large database to smaller, more accessible subsets, many potentially useful compounds and novel or unconventional chemotypes could be overlooked. It is essential to maximize the number of database entries tangibly evaluated against a target of interest. Additionally, a vast majority of docking data is not being utilized while it could represent a very relevant, well-formatted, and content-rich landscape for machine learning algorithms. Typical docking campaigns rely on completing a full docking run and selecting only an extremely narrow subset (~1%) of favorably docked molecules for future evaluation.

Ultra-large VS in ion channel drug discovery offers numerous advantages compared to conventional experimental high throughput screening methods. VS enables the exploration of a vast chemical space, including millions of potential ion channel modulators, increasing the likelihood of discovering novel hit compounds with unique structures. It is cost and time-efficient, allowing for rapid evaluation of compounds *in silico*, reducing the need for expensive and time-consuming experimental synthesis and testing. VS enables early hit identification, helping researchers prioritize the most promising compounds and saving resources by excluding less viable candidates. It provides mechanistic insights into ion channel interactions and facilitates rational design and optimization of modulators. Additionally, VS allows evaluation of rare or challenging-to-source compounds, such as natural products or derivatives, enhancing the probability of discovering valuable hits. VS serves as a complement to experimental screening, and computational predictions can guide subsequent experimental validation and optimization of identified hits.

In this review we will introduce basic principles of VS and methodology behind it. We will give an overview of the knowledge base of ion channel structures and how they can be generated. We will describe chemical libraries of small molecules that are used to screen ion channel structures and we will go through several real VS campaigns on ion channels utilizing different docking-based, ligand-based, and deep learning VS techniques.

## Overview of virtual screening methods

Ultra-large VS technologies refer to computational methods and techniques used to screen large chemical libraries against a target of interest in drug discovery. VS is a firmly established technique in computational drug design which can greatly reduce the costs of discovering a new drug. In general, VS aims to identify potential drug candidates by simulating and predicting their interactions with a target protein or biological system. Ultra-large VS technologies take this concept to a larger scale, enabling the screening of massive chemical libraries containing millions or even billions of compounds. The methodology of ultra-large VS will be reviewed below in the sections devoted to each of the methods and their applications in ion channel drug discovery campaigns will be illustrated. The primary goal of ultra-large VS is to narrow down the chemical space and prioritize the most promising compounds for further experimental validation. It is an important tool in the early stages of drug discovery where large-scale screening can significantly reduce the time and cost associated with traditional high-throughput screening methods.

**Figure 1:**
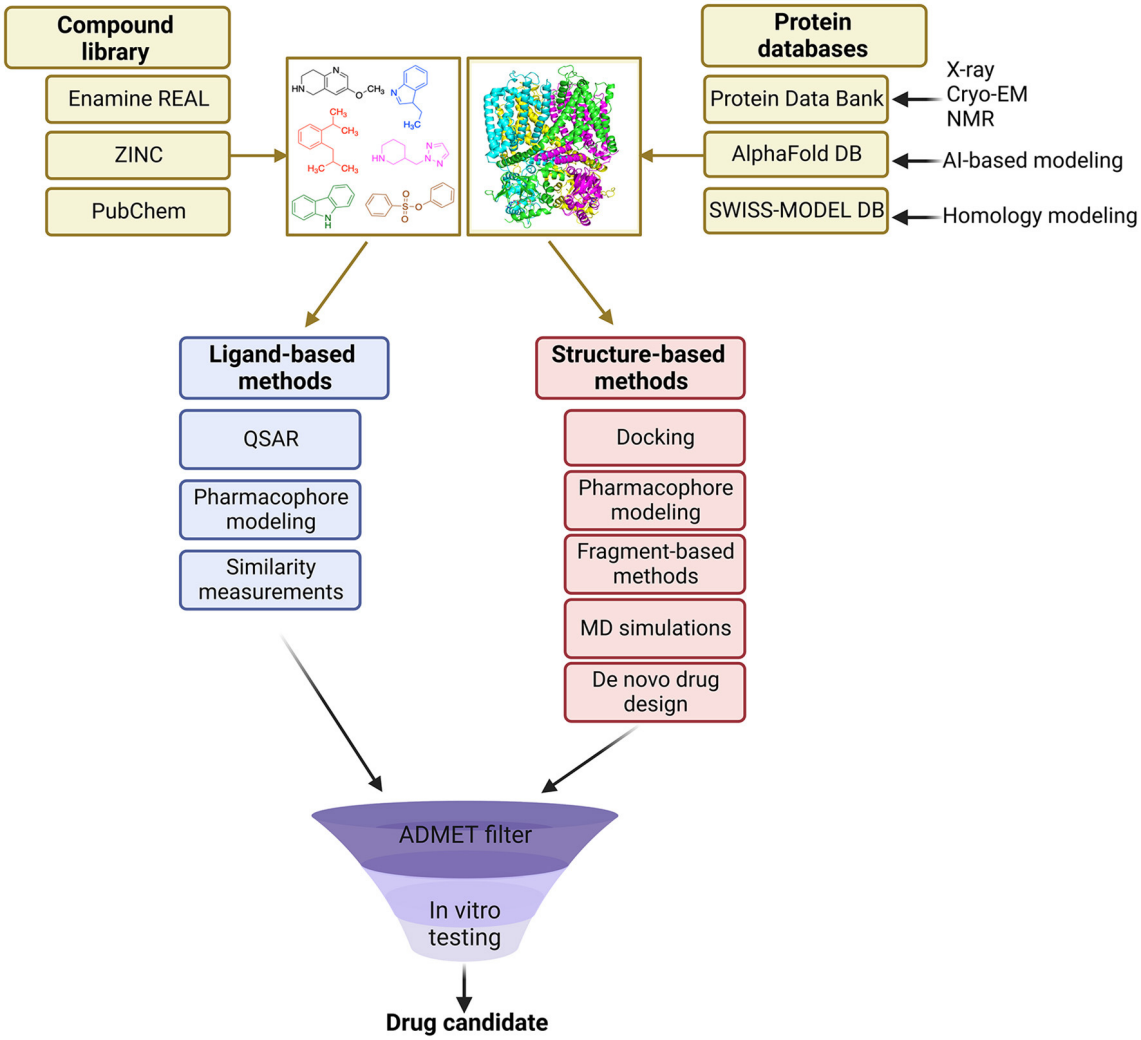
Overview of the general VS workflow.

VS methods can be divided into two main categories: ligand-based and structure-based approaches (Figure 1). Ligand-based methods rely on the similarity of identified molecules to the known actives, whereas structure-based methods aim to predict the binding pose of molecules based on the known 3D protein target (Lavecchia and Di Giovanni, 2013).

Ligand-based VS (LBVS) approach is less computationally expensive than the structure-based method because it does not rely on macromolecular structure in calculations. LBVS methods are usually performed by comparing fingerprints of tested molecules with the ones from the active set. These fingerprints can be of various types, such as topological descriptors, circular and pharmacophore-based fingerprints, etc. Similarity searching and quantitative structure-activity relationship (QSAR) modeling can be applied to compare the fingerprints and derive the underlying relationship between molecules and their activities (Gimeno et al., 2019).

The analysis and recognition of QSAR has also become an essential component of ligand-based VS techniques and the pharmacology of ion channels. QSAR is an attempt to establish a correlation between the chemical structure of a molecule and the biological effect. The representation of chemical structures can be described through molecular descriptors: 1-D descriptors encode generic properties such as molecular weight, hydrophobic/hydrophilic partition coefficient, and molar refractivity, commonly related to a basic description of drug-likeness; 2-D descriptors predict physicochemical properties, and provide quantitative estimates of biological activity from topological representations of the molecules; 3-D descriptors are derived as the name implies, from the 3-D structures of the molecules, depending on the conformation used and the flexible superposition of the molecules. 3-D QSAR offers a better representation of molecules interacting with proteins and leads to statistically improved models. QSAR analysis builds on mathematical models, e.g., random forest, decision trees, naive Bayes classifier, support vector machines, k-nearest neighbors, and artificial neural networks, to find some statistical correlation between the biological parameters of tested molecules derived from various assays (pEC_50_, Ki, activity, toxicity, etc.) and their chemical structures (Hansch and Fujita, 1953). Regression and classification techniques are applied to derive the relationship between molecules, which can be substantiated by machine learning (Neves et al., 2018) and pharmacophore modeling.

Structure-based VS (SBVS) methods require a 3D structure of a protein of interest, and tested molecules are ranked according to their activity toward a receptor obtained from calculations (Maia et al., 2020). Docking is the main approach used in SBVS (Kuntz et al., 1982), which can be also supported by machine learning to derive scoring functions, which evaluate the binding orientations of molecules, as well as deep learning that speeds up the docking protocol and allows to screen billions of molecules in much shorter time (Pereira et al., 2016; Gentile et al., 2022). SBVS can also utilize pharmacophore modeling, which does not focus on a specific ligand structure, but rather defines necessary functional groups that a molecule should possess to create interactions in a receptor's binding pocket (Giordano et al., 2022). Another approach in SBVS is fragment-based virtual screening, which involves screening small molecular fragments against a target protein, followed by growing, merging or linking fragments into larger drug-like molecules (Doak et al., 2016). Since the key aspect of fragment-based VS requires the availability of the 3D structure of the protein target, this technology is directly related to SBVS (Murray and Rees, 2009). By employing fragment docking or fragment-based *de novo* design techniques, millions or billions of molecules can be computationally screened against a target. One advantage is that the small size of the fragments allows a more efficient search of chemical space and recovers more protein binding information than in traditional high-throughput screens, allowing the size of the library to be much smaller. Furthermore, the combination of fragment-based and combinatorial chemistry approaches allows designing target-focused and diverse chemical libraries (Liu et al., 2017). Hits obtained from this screening can be expanded and optimized to develop more potent drug candidates (Li, 2020).

In the following sections we will introduce the concepts of several VS methods and review their applications in recent ion channel drug discovery campaigns.

## Ion channel structures for virtual screening

**Figure 2:**
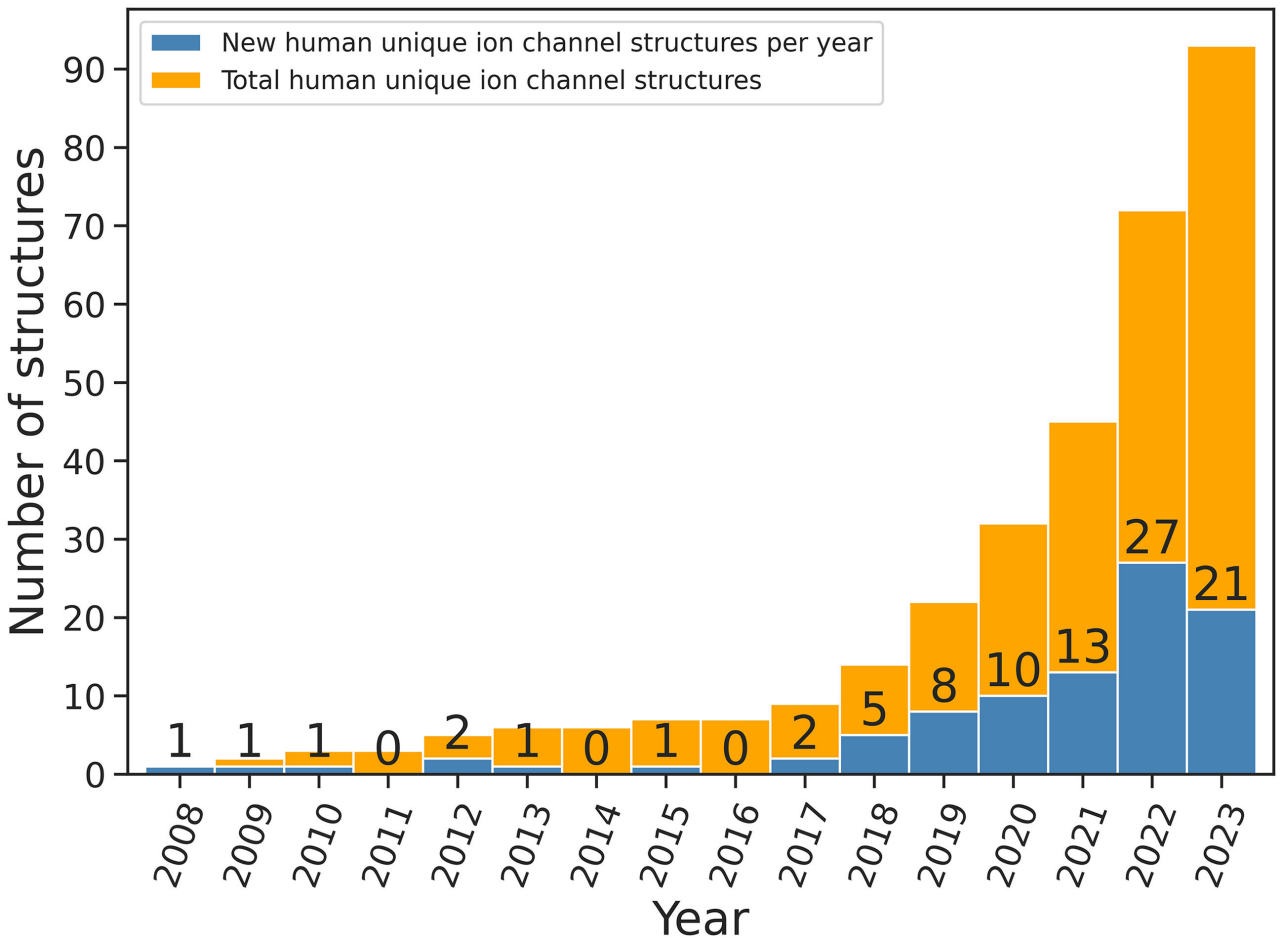
Number of unique structures of human ion channels released every year since 2011 (data were obtained from mpstruc database, source: mpstruc database: Available at: https://blanco.biomol.uci.edu/mpstruc/, accessed on 23 October 2023). Values on top of blue bars indicate the number of new human ion channel structures released every year.

Ultra-large VS efforts benefit from the quickly growing number of protein target structures. For instance, for the largest group of drug targets, G protein-coupled receptors (GPCRs), VS experiments have flourished because of a larger number of available GPCR structures (Luttens et al., 2022; Matricon et al., 2023). Similarly, the number of ion channel structures has increased tremendously over the last 25 years; from the determination of the first ion channel structure, the KcsA channel from Streptomyces lividans, in 1998 (Doyle et al., 1998) to more than 1,500 structures nowadays. This includes ca. 200 ion channel structures from the human proteome, with almost 100 of them being unique human ion channel structures (Figure 2) (Pliushcheuskaya and Künze, 2023). This progress was primarily due to technological advancements in X-ray crystallography and cryo-EM.

If an experimental structure for an ion channel of interest is lacking, structure prediction methods like AlphaFold (Jumper et al., 2021), RoseTTAFold (Baek et al., 2021), or ESMFold (Lin et al., 2023) offer a solution. For instance, The AlphaFold database estimates more than 290 non-redundant structures for ion channels for the human proteome (Varadi et al., 2022). These artificial intelligence-based prediction methods can provide highly accurate model structures which are often suitable for VS applications.

**Figure 3:**
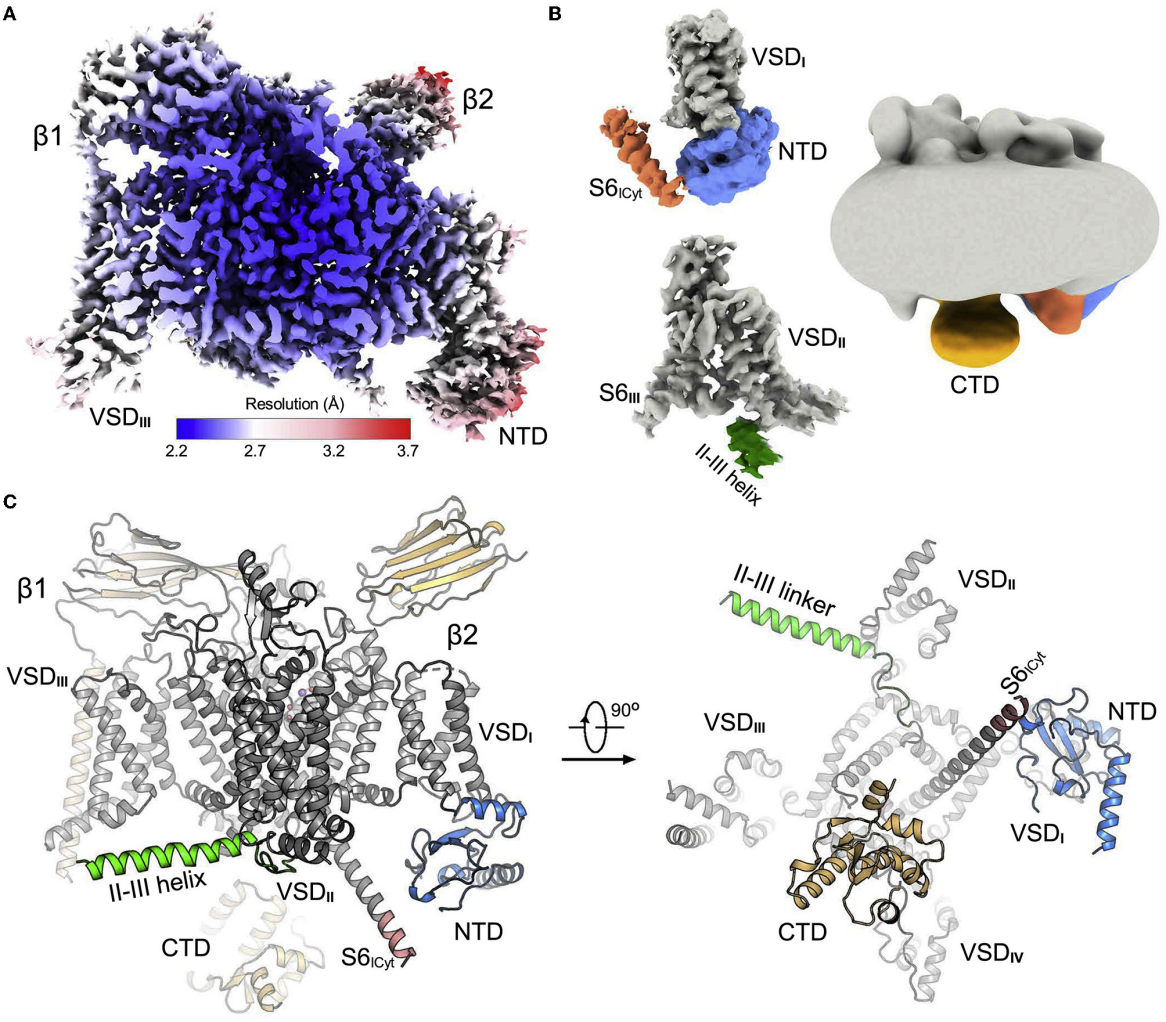
Cryo-EM structure of wild-type human Na_V_1.7. **(A)** EM map of wild-type Na_V_1.7-β1-β2 complex. **(B)** EM maps of previously unresolved cytosolic regions. **(C)** Structure of Na_V_1.7-β1-β2 complex. The core domain is gray, previously unresolved regions are colored correspondingly to those in **(B)**. VSD, voltage-sensing domain; NTD, amino-terminal domain, CTD, carboxy-terminal domain. Reprinted (adapted) from Huang et al. (2022a). Copyright 2022, Elsevier.

Another use case of AlphaFold structure prediction is to aid interpretation of low-resolution electron density maps of ion channels. For instance, Huang et al. (2022a) studied the voltage-gated sodium ion channel Na_V_1.7, which is highly expressed in nociceptive neurons and is a drug target for pain relief (Hameed, 2019). Specifically, structures of Na_V_1.7 in combination with pore blockers are of high interest to better understand the mechanism of Na_V_1.7 modulation and develop effective analgesics (Zhang et al., 2022b). In the study of Huang et al. (2022a), various wild-type and mutant structures of Na_V_1.7 with and without small-molecule ligands were determined. The use of AlphaFold was key to facilitate interpretation of intracellular low-resolution regions of the cryo-EM map, which are of direct interest for designing inhibitors of Na_V_1.7. Eventually, the authors were able to identify determinants of the Na_V_1.7 channel modulation, which was enabled by the accurate structure determination of Na_V_1.7 (Figure 3).

## Chemical libraries for ultra-large virtual high-throughput screening

Physically available compound collections in academic and industrial research institutions typically range from thousands to millions of compounds (Sadybekov and Katritch, 2023). In contrast, virtual compound libraries can be much larger, reaching billions of compounds, with the aim of enhancing chemical diversity coverage (Grygorenko et al., 2020; Huang et al., 2022b; Kuan et al., 2023; Lyu et al., 2023). Chemical space encompasses all possible organic molecules, estimated to be around 10^60^ or more. This vastness offers an opportunity to discover unique biological activities and mechanisms of action not found in general screenings.

Enamine is a chemical supplier that offers diverse small molecule collections for screening and hit expansion, focusing on synthetically accessible molecules. The REAL (Readily Accessible) database, popular among researchers, contains over 38 billion compounds with varied chemical scaffolds (REAL Compounds, n.d.). Widely used in drug discovery stages such as hit identification, lead optimization, and fragment-based screening, the REAL library is a significant advancement in combinatorial chemistry. It features simple fragments and building blocks connected through efficient one- or two-step reactions, allowing for a vast number of unique combinations. This versatility makes it valuable for diverse molecular structure generation in VS and positions it as a crucial resource in drug discovery and material science. Similar extensive libraries are offered now also by other vendors like eMolecules (eMolecules, n.d.).

The ZINC database is a widely used resource that provides a vast collection of commercially available compounds for VS and drug discovery. It contains over 230 million small molecules with diverse chemical structures (Irwin and Shoichet, 2005), of which approximately 34 million predictions belong to the ion channel major class. Regarding the number of reactions, the ZINC database primarily focuses on providing commercially available compounds, while the Enamine REAL database emphasizes synthetically accessible compounds. As a result, Enamine's REAL database may have a larger number of enumerated reactions available for the compounds within its library (Saldívar-González et al., 2020). However, it is worth noting that the exact number of compounds in each database can vary over time due to updates and additions.

PubChem is a free chemical database maintained by the National Center for Biotechnology Information (NCBI). It offers a vast collection of chemical substances (over 293 million), small molecules (over 110 million), bioassays (over 1.25 million), and their associated data (Kim et al., 2016). PubChem is extensively used in drug discovery research. PubChem primarily focuses on aggregating and curating chemical data from various sources, while the Enamine REAL database specifically emphasizes synthetically accessible compounds.

ChEMBL is a large database of over 2.4 million bioactive compounds and their associated biological activities (Gaulton et al., 2012) where approximately 190,000 are associated with an ion channel family major class. It provides access to chemical structures, target information, and bioactivity data extracted from scientific literature. ChEMBL is commonly used for drug discovery, lead optimization, and target identification. It primarily focuses on capturing and curating bioactivity data and target information rather than enumerating chemical reactions.

Another type of VS libraries are combinatorial libraries which consist of related molecules systematically generated by altering structural scaffolds or through parallel synthesis methods. These libraries efficiently explore chemical space and save time in drug discovery and other applications. Scaffold hopping and reaction-based scheme are two common approaches to create such libraries, enabling the generation of structurally related compounds with potential variations in biological activity. By defining the main substituents that molecules should possess, combinatorial libraries can be generated by a scaffold mining with addition of these substituents to varying chemical cores (Varin et al., 2011; Hu et al., 2017). This approach is usually applied when a specific protein target is known and pharmacophoric elements are of importance for a potential drug to be active (Karthikeyan et al., 2015). The other way of generating combinatorial libraries involves defining a set of chemical reactions that should be followed in order to generate final molecules by connecting building blocks (Podlewska et al., 2017; Suay-García et al., 2022). Several pharmaceutical companies develop their own reaction-based combinatorial libraries, such as Eli Lilly's Proximal Collection (Nicolaou et al., 2016) and Pfizer global virtual library (Hu et al., 2012).

There are also ion channel-focused combinatorial libraries, like the IONCore library developed by ChemBridge (n.d.). This is an ion channel-focused library consisting of ca. 6000 small molecules, which were compiled based on 3D similarity to published compounds with activity against ion channels. SelleckChem developed an ion channel ligand library consisting of 745 small molecules to target a diverse set of ion channels (Ion Channel Ligand Library, n.d.). Furthermore, Enamine provides an ion channel-targeted library containing ca. 40,000 compounds, subdivided into collections for major ion channel families, e.g., the calcium ion channel library with 10,560 compounds and the sodium ion channel library with 5440 molecules, respectively (Ion Channel Library, n.d.).

Other commercial vendors provide chemical additional databases, e.g., MolPort (n.d.). MolPort aggregates compounds from various suppliers and contains ca. four million entities, including molecules generated via combinatorial methods and reaction-based approaches.

## Challenges of CADD on ion channels compared to soluble protein targets

CADD encounters distinctive challenges when targeting ion channels in comparison to soluble protein targets. The lipid membrane environment, in which ion channels reside, poses complexities in accurately predicting ion channel-ligand interactions due to the unique physicochemical properties of the membrane and additional interactions that are formed between the small molecule compound and lipids. Furthermore, the involvement of ion channels in regulating ion concentration gradients and changing the electrical field properties across the membrane emphasizes the significance of considering electrostatic interactions for modeling ligand binding. Additionally, the narrow and deep binding sites in ion channels, particularly in the pore region, present challenges in designing ligands that can efficiently navigate these confined spaces. This can be further complicated by the fact that ion channels switch between multiple conformational states during gating and that experimentally determined structures are available for only a handful of these states, also underscoring the intricacies of CADD in this context. Growing evidence from biophysical and structural investigations suggest association of many small-molecule drugs with the membrane-exposed surface of ion channels (Payandeh and Volgraf, 2021). The absence of a direct path from bulk solvent to the binding site entails an initial partitioning into the membrane, fundamentally shaping the drug's interaction with the protein target. This membrane access mechanism imparts a critical influence on potency data, structure-activity relationships, pharmacokinetics and physicochemical properties.

MD simulations can be an accurate method for simulating ion channel-ligand binding events within the membrane region (Gumbart et al., 2005; Goossens and De Winter, 2018), because the dynamic behavior of both the protein and the surrounding lipid bilayer can be explicitly modeled. While MD approaches can provide valuable insights, they are usually time-consuming, limiting their applicability to a small number of ligands. The intractability of MD for extensive ligand sets has led to the exploration of alternative techniques such as free energy perturbation (FEP) (Kuhn et al., 2020). In a recent study, Dickson et al. (2021), applied FEP to calculate the relative binding energies of a series of antagonists that target the lipid-exposed, extra-helical site of a membrane protein. By constructing an appropriate thermodynamic cycle, the authors were able to uncouple the membrane partitioning of the drug from the drug binding at the lipid-exposed site and could calculate the free energy for each step. Because of its promising performance the protocol might be applied in a predictive manner on larger datasets of ligands targeting protein-membrane interfaces. This approach holds potential for enhancing the efficiency and scalability of computational studies focused on membrane protein-ligand interactions.

The hydrophobic environment of the lipid bilayer poses a challenge for CADD due to its impact on the energetics of ligand binding. The dynamics of the lipid bilayer further complicates matters, influencing conformational changes in ion channels, which are crucial for their function. Incorporating these aspects into computational protocols presents a complex task, requiring ongoing computational protocol development and optimization of force fields. One such specialized protocol was devised within the Rosetta framework for the docking of cholesterol to integral membrane proteins (Marlow et al., 2023). The so-called RosettaCholesterol protocol, based on RosettaLigand (Meiler and Baker, 2006), adapts the sampling and scoring steps to improve docking of the cholesterol ligand and adds an additional filtering step to predict the cholesterol binding site specificity. The RosettaCholesterol protocol improved sampling and scoring of native poses over the RosettaLigand baseline in 91% of cases. Furthermore, the authors were able to recapitulate experimentally validated specific sites on the β2 adrenergic receptor. It proves to be a computationally fast and inexpensive tool that can screen many possible protein-cholesterol complexes. Future studies may further refine Rosetta-based protocols to explore a broader spectrum of lipid-protein interactions, paving the way for a more comprehensive understanding of membrane biology and protein-lipid dynamics.

Some lipids also have pharmacological effects and can be utilized to inform drug design. Notably, polyunsaturated fatty acids (PUFAs) can serve as signaling molecules with pharmacological effects, influencing cellular processes and modulating ion channels and inflammation (Xiao et al., 2005). Understanding the roles of bioactive lipids in cellular signaling informs the rational design of compounds targeting the protein-lipid surface. For example, Yazdi et al. (2021) studied PUFAs-modulated activation and mode of binding on KCNQ1 channels. Utilizing MD and electrophysiological experiments, they observed that PUFAs bind to the KCNQ1 voltage sensor and pore domain. The positively charged amino acid residues in these regions favorably stabilize the electronegative head group of PUFAs, while the tail group maintains the open position of KCNQ1 upon interaction with the hydrophobic residues. Different PUFA analogs produce a range of modulatory effects in ion channels (Bohannon et al., 2020a,b) which can be a useful information to guide the design of anti-epileptic and anti-arrhythmic drugs.

There exists still much uncertainty about the location of possible druggable sites in ion channels and new binding sites have been often discovered at unexpected locations (Wright et al., 2020; Sridhar et al., 2021; Botte et al., 2022; Kschonsak et al., 2023). Hence, methods that can accurately predict ligand binding sites on membrane proteins will significantly improve drug discovery. Lu et al. (2019) described a machine learning-based classifier tailored to the prediction of ligand binding sites on membrane protein surfaces. The MPLs-Pred method uses evolutionary profiles, topological features, physicochemical properties, and primary sequence segment descriptors as combined features in a random forest classifier. MPLs-Pred achieved an appreciable performance with Matthew's correlation coefficients of 0.597 and 0.356 on cross-validation and independent test sets, respectively. Ligand-specific predictive models that classify ligands into drugs, metal ions and biomacromolecules further improved the prediction performance. Notably, the versatility inherent in the approach above suggests the potential for its extension to accommodate the prediction of various other ligand species.

The characteristics of ion channel binding sites, particularly the narrow and deep pores, requires molecules that can efficiently navigate and bind within these confined spaces. CaverDock, developed by Vavra et al. (2019), is a docking tool based on AutoDock Vina (Eberhardt et al., 2021) which can simulate the binding and unbinding of ligands to protein tunnels like ion channel pores. This tool uses the optimized docking algorithm of AutoDock Vina for ligand placement and implements a parallel heuristic algorithm to search the space of possible trajectories. In comparison with MD simulations, CaverDock does not require extensive knowledge of the studied system. CaverDock can sample the binding energy throughout the whole protein tunnel and identify unfavorable binding interactions, which can then be optimized by site-directed mutagenesis.

Ligand docking scoring functions used in CADD may be less accurate for membrane proteins because of the unique physicochemical environment of lipid membranes and because scoring functions have been usually optimized for soluble protein-ligand systems (Li et al., 2019; Rudden and Degiacomi, 2021), emphasizing the need for refinement and validation of scoring approaches tailored to ion channels. The presence of charged residues in the membrane surface region necessitates careful consideration of electrostatic interactions. Therefore, tuning the scoring function may involve emphasizing terms related to electrostatic forces, including charged interactions between the ligand and the protein. This is also observed in the hydrophobic deep membrane region, where the lipid bilayer provides a nonpolar environment, requiring adjustments of the solvation and electrostatic score terms. Unfortunately, the availability of tools tailored for ligand docking scoring functions is relatively limited, with a predominant focus on the development and benchmarking of ligand docking scoring functions for soluble receptors (Li et al., 2019). Very few docking programs have incorporated membrane scoring functions to address the challenges associated with modeling interactions at the protein-membrane interface. Usually these computational frameworks are intended for protein docking but can be adapted to ligand docking as well. MEMDOCK is an algorithm designed specifically for docking alpha-helical membrane proteins within the membrane. The method models both side chain and backbone flexibility and performs rigid body optimization of the ligand orientation using modified Patchdock and Fiberdock (Hurwitz et al., 2016). Furthermore, HADDOCK (Dominguez et al., 2003), LightDock (Jiménez-García et al., 2018), and Rosetta (Leman et al., 2020) also offer the possibility for protein and ligand docking in an implicit membrane model. Within the Schrödinger suite of tools, Glide (Halgren et al., 2004) and Desmond (Desmond Software, n.d.) allow for an integrated workflow in which researchers can first conduct MD simulations on membrane proteins using Desmond and subsequently transition the resulting structures to Glide for ligand docking experiments. This offers a holistic exploration of membrane protein dynamics and facilitates a detailed examination of ligand binding within the context of the lipid bilayer environment. While other common docking programs such as ZDOCK (Pierce et al., 2014) and AutoDock (Eberhardt et al., 2021) traditionally lack built-in membrane protein scoring functions for ligand binding, noteworthy adaptations and integrations have been introduced to enable their functionality in the context of docking ligands into transmembrane domains (Greene et al., 2016; Kobeissy Stanley M Stevens and editors, 2017). These modifications often involve specialized considerations for the hydrophobic and electrical properties of membrane environments.

## Docking-based ultra-large virtual screening

In docking-based VS a panel of protein targets is screened with various molecular docking software to model the binding mode and interactions with small molecules in the binding pocket. The top-ranked small molecules are prioritized for further studies (Lazar et al., 2017). Software tools and platforms like AutoDock, DOCK, Glide, DiffDock, and Deep Docking (DD) facilitate high-throughput docking by employing diverse scoring functions and algorithms to predict compound binding affinity and orientation within the target's binding site (Kuntz et al., 1982; Halgren et al., 2004; Trott and Olson, 2009; Gentile et al., 2020; Corso et al., 2022).

Hughes et al. (2019) applied docking-based VS to discover new modulators of transient receptor potential vanilloid 5 (TRPV5) ion channels. TRPV5 is a calcium-selected ion channel, which plays an important role in renal calcium homeostasis in the human organism (Dang et al., 2019). TRPV5-knockout mice were shown to exhibit hypercalciuria and nephrolithiasis proving its critical role in calcium levels maintenance (De Groot et al., 2008). There are several existing TRPV5 modulators (Nilius et al., 2001; Hughes et al., 2018), but they lack selectivity over TRPV6 subtype, which is the closest homolog of TRPV5. In this regard, Hughes et al. (2019) conducted a VS experiment at the inhibitor binding site of TRPV5 utilizing the ZINC15 library with over 12 million molecules. The library was docked into TRPV5 using the Glide software package from the Schrödinger suite (Friesner et al., 2004), and top-100 best decoys were clustered into 65 groups with unique scaffolds based on the Tanimoto similarity score. 43 were proceeded to physiological testing by means of whole cell patch clamp experiments. Several compounds from this set were shown to inhibit TRPV5-induced currents, with two of them, ZINC9155420 and ZINC17988990, exhibiting additionally selectivity for TRPV5 over TRPV6. The authors also determined cryo-EM structures of these two ligands bound to the TRPV5 channels and identified new binding sites that provided insights into the ligand mode of specificity.

Wacker et al. (2012) studied heteromultimeric K_V_ channels in VS experiments. Particularly, K_V_1.1–1.2 represents the most abundant potassium channel multimer in central and peripheral nervous systems (Coleman et al., 1999). K_V_1.1–1.2 channels are highly expressed in the hippocampus and are an important target in epileptic seizures and multiple sclerosis (D'adamo et al., 2020). The known K_V_1.1–1.2 inhibitor, 4-aminopyridine, however, has limited potency and also inhibits other K_V_ channels (K_V_1.4, K_V_4.2 subtypes), which compromises cardiac safety (Goodman et al., 2009, 2010). Wacker et al. (2012) conducted docking-based VS with Autodock-Vina (Trott and Olson, 2009) on K_V_1.1-1.2 using the ZINC library containing ~10 million molecules. From 200 top scored compounds 89 compounds were tested using patch clamp experiments. Fourteen of 89 compounds showed some inhibitory activity ranging from 0.6 to 6 μM on K_V_1.1–1.2 channels (compared to 4-aminopyridine with IC_50_ = 170 μM on K_V_1.1 and IC_50_ = 230 μM on K_V_1.2), and two of them also showed a higher potency toward inhibition of K_V_1.1–1.2 in respect to other channels (hERG, Ca_V_1.2, Na_V_1.5).

Docking-based VS was also utilized in the study of Oddsson et al. (2020), aiming at identifying new dual target molecules against Alzheimer's disease, acting via nicotinic acetylcholine receptors (nAChRs). The same research group showed that a combination acetylcholinesterase (AChE) inhibitors and activators of nAChRs can lead to beneficial effects in the symptomatic treatment of Alzheimer's disease (Zoli et al., 2015; Kowal et al., 2019). The research group speculated that the increased activity of α7 nAChR, which is a ligand-gated ion channel, may improve treatment in Alzheimer's disease. Correspondingly, Oddsson et al. (2020) performed VS on both target proteins using the ZINC15 dataset with a total number of ~four million compounds. All molecules were docked into AChE and nAChRs, and from the top-scored ligands in both proteins a common subset was selected that was encountered in both screening runs. One of the identified hit molecules showed the desirable inhibitory effect on AChE and agonistic activity on nAChR when evaluated in voltage-clamp electrophysiological testing. The identified compound represented the first example of a multitarget compound for the treatment of Alzheimer's disease.

Additional notable docking-based VS studies on ion channels include the discovery of small-molecule activators of the KCNQ1 channel (Liu et al., 2020; Lin et al., 2021) and of allosteric modulators of BK channels (Zhang et al., 2022a). All of these studies used the MDock docking software (Yan and Zou, 2015) and the Available Chemical Database (ACD) for screening. Interestingly, the identified compounds were found to affect their ion channel targets via different modes of action. The KCNQ1 activator molecule CP1 mimics the lipid PIP2 in mediation of voltage sensor-pore coupling and thereby enhances KCNQ1 activation (Liu et al., 2020). C28 is another KCNQ1 activator molecule but binds to and stabilizes the voltage sensor domain, thereby decreasing the voltage required for voltage-dependent KCNQ1 activation (Lin et al., 2021). The authors found that C28 can effectively reverse drug-induced lengthening of the action potential duration in ventricular myocytes. The small-molecule BK channel allosteric modulator BC5 binds at the voltage sensor-cytosolic tail domain interface and specifically enhances Ca^2+^-dependent activation by perturbing the pathway for coupling between Ca^2+^ binding and pore opening (Zhang et al., 2022a). This mode of action was corroborated by mutagenesis and atomistic simulations and suggested that the interface between voltage sensor and cytosolic tail domain in BK channels is an important site for allosteric modulation.

Ultra-large VS requires fast algorithms that are able to predict the activity or binding affinity of billions of compounds in reasonable time. Deep Docking (DD), is a novel deep learning platform that is suitable for docking billions of molecular structures in a rapid, yet accurate fashion (Gentile et al., 2020). The DD approach employs sophisticated deep neural network models rooted in QSAR principles. These models are trained using docking scores from a small subset of a molecule library. The primary goal is to predict the docking results for new entries and iteratively exclude unfavorable molecules. By integrating the DD methodology with the FRED docking program, Gentile et al. achieved rapid and precise computation of docking scores for 1.36 billion molecules sourced from the ZINC15 library (Gentile et al., 2020). This extensive analysis covered 12 notable target proteins. Notably, this approach led to an impressive data reduction of up to 100-fold and a remarkable enrichment of high-scoring molecules by a factor of 6,000. Importantly, these advancements were attained without any substantial loss in the successful docking of molecules. The DD protocol can be seamlessly incorporated into many docking programs and is publicly available (Gentile et al., 2020).

In a recent study conducted by Yang et al. (2023), the researchers utilized deep docking-facilitated VS in conjunction with the VirtualFlow platform to screen ligands targeting the inward rectifier potassium channel 5.1 (Kir5.1, KCNJ16). VirtualFlow is a flexible and parallel workflow platform designed to execute VS tasks on Linux-based computer clusters of various sizes and types, all seamlessly managed by a batch system. The author's gene profiling and enrichment analyses revealed that KCNJ16 exhibited downregulation in thyroid tumor tissues compared to normal ones, implicating a pivotal role for KCNJ16 in cell growth and differentiation. Consequently, Kir5.1, encoded by KCNJ16, emerged as an appealing target in thyroid cancer research. To narrow down their selection of compounds, Yang et al. employed the DD protocol and executed the final docking run using VirtualFlow. They relied on the AlphaFold predicted structure of Kir5.1 for docking. Employing the DD protocol, the authors identified several molecules, including Z2087256678_2, Z2211139111_1, Z2211139111_2, and PV-000592319198_1, as potent ligands for Kir5.1. Unfortunately, the computationally identified hit molecules were not further tested and, therefore, should be regarded as suggestive.

V-SYNTHES (virtual synthon hierarchical enumeration screening), developed by Sadybekov et al., represents another approach aimed at efficiently screening ultra-large compound libraries for potential hit compounds using a modular synthon-based strategy (Sadybekov et al., 2022). Essentially, a synthon represents a fragment of a molecule that can be used as a building block for synthesizing more complex molecules. In this pioneering study, the algorithm enabled efficient screening of the Enamine REAL library and its REAL Space extension containing over 11 billion drug-like compounds.

**Figure 4:**
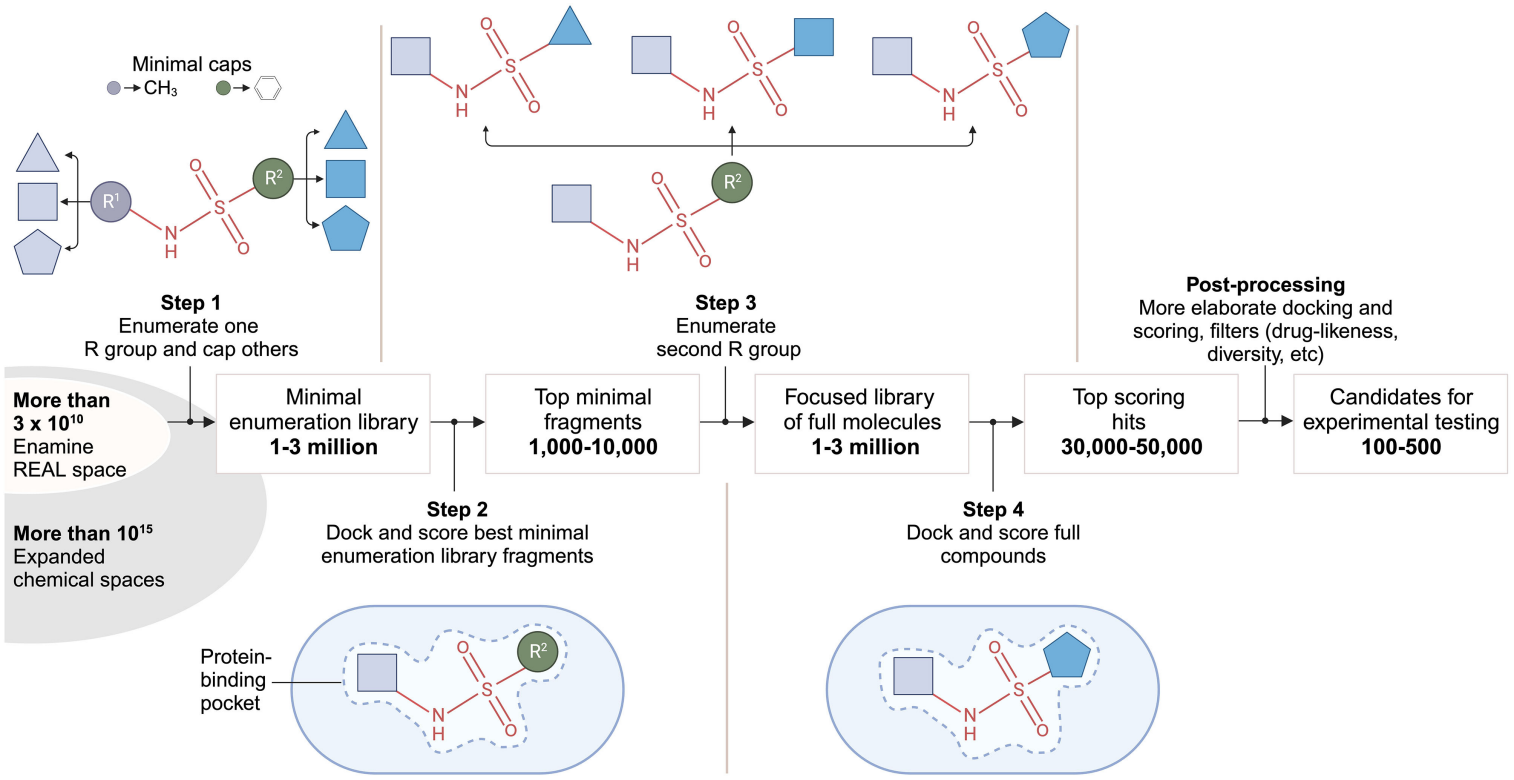
V-SYNTHES approach to modular screening of Enamine REAL Space. The flow chart from left to right provides a broad outline of the 4-step algorithm developed by Sadybekov et al. (2022).

V-SYNTHES involves several iterative steps (Figure 4), starting with the creation of a fragment-like compound library representing all possible scaffold-synthon combinations. In the first step, compounds are constructed by combining reaction scaffolds with corresponding synthons at one position, while other positions are capped. Capping refers to the process of modifying or blocking certain positions on a molecule while allowing chemical reactions to occur at specific, chosen positions. This results in a library of ~600,000 compounds, corresponding to the number of synthons. In the second step, docking simulations are used to predict the binding affinity of these fragments to a target protein. The top-scoring candidates (1,000–10,000) undergo further rounds of enumeration and docking in steps three and four. The final set of 50–100 compounds for experimental testing is selected based on post-processing filters e.g., synthesizability.

In the original V-SYNTHES paper (Sadybekov et al., 2022), the Enamine REAL Space library was screened for cannabinoid receptor antagonists using a receptor-template-based approach. V-SYNTHES significantly accelerated the screening process, requiring docking of only ca. 2 million compounds, while screening a much larger chemical space. Furthermore, V-SYNTHES outperformed traditional brute force VS approaches by identifying more high-scoring compounds. The approach yielded 80 hit candidates, with 60 synthesized and functionally characterized. Notably, 33% of these hits had K_i_ values better than 10 μM. This hit rate was twice as high as the hit rate achieved in standard docking of a representative subset of the Enamine REAL library.

The hit compounds showed diverse structures, containing new scaffolds and fully occupying the receptor's orthosteric binding pocket. Application of V-SYNTHES to the kinase target ROCK1 also led to successful results, with a 28.5% hit rate containing compounds with nanomolar affinity. Overall, V-SYNTHES provides a practical and efficient method for rapidly screening ultra-large modular virtual libraries. It can be adapted to various docking-based screening platforms and applied to ion channels and other target proteins, which demonstrates the broad applicability of the method in drug discovery efforts.

## Ligand-based virtual screening

Ligand-based VS techniques play a crucial role in the field of ion channel drug discovery by aiding in the identification of potential compounds that could modulate ion channel activity. Ligand-based VS focuses on identifying molecules with structural and chemical properties similar to known ion channel modulators. This approach is particularly useful when experimental structural information about the target ion channel is limited or unavailable. The ligand-based methods include approaches like similarity and substructure searching, QSAR modeling, pharmacophore-based search, and 3D shape matching supported by machine learning and molecular modeling techniques.

Ligand-based VS enables the efficient exploration of large compound databases, identifying molecules that exhibit potential ion channel modulatory effects. This approach helps prioritize compounds for experimental testing, reducing the time and resources required in early stages of drug discovery. Overall, ligand-based VS techniques complement experimental approaches in ion channel drug discovery and contribute significantly to the identification of novel therapeutic candidates targeting ion channels, potentially leading to the development of innovative treatments for various diseases (Sharma et al., 2021).

Ijjaali et al. (2007) focused on the use of ligand-based VS techniques for the discovery of novel T-type calcium channel inhibitors. T-type calcium channels are implicated in various neural disorders such as epilepsy and neuropathic pain. To identify new inhibitors, the researchers employed a pharmacophore-based VS approach using 2D pharmacophoric fingerprints. They collected a dataset of known active compounds from the AurSCOPE Ion Channels knowledgebase (AurSCOPE Ion Channel Database, n.d.), which was used as a query to screen an external molecular database. A total of 38 compounds were selected for biological evaluation, and functional patch clamp assays were conducted on the Ca_V_3.2 isoform. Interestingly, 16 out of the 38 compounds showed more than 50% blockade of Ca_V_3.2-mediated T-type current. These findings demonstrate the effectiveness of ligand-based VS in identifying potential T-type calcium channel inhibitors for further investigation in drug discovery efforts.

Mohan et al. conducted a study aiming to identify compounds with N-type calcium channel blocking activity (Mungalpara et al., 2010). They utilized multiple descriptors such as structure, ADME/Tox, thermodynamics, and electrotopological properties to train a QSAR model for predicting blocking activity. The resultant descriptors offered insights into the physico-chemical attributes influencing N-type calcium channel blocking activity.

The team led by Noskov evaluated a collection of hERG pore domain blockers through a combination of 3D-QSAR and receptor-based molecular docking techniques (Durdagi et al., 2011). They also designed a pharmacophore model that enabled swift assessment of compound channel-blocking capability. The outcomes were corroborated by docking hits into a hERG homology model and through *in silico* mutagenesis, aligning closely with experimental data.

Pharmacophore-based screening methods represent another powerful approach for ligand-based VS. Pharmacophore models represent the critical chemical features of the ligand molecule and their spatial arrangement required for compound-target interactions. These methods can swiftly search large chemical databases for compounds that conform to the pharmacophoric constraints of the target (Urbahns et al., 2003; Dror et al., 2009; Seidel et al., 2010; Giordano et al., 2022). Pharmacophore-based screening is particularly useful when the protein target structure is challenging to obtain (Kaserer et al., 2015; Schaller et al., 2020).

Sehgal et al. (2014) performed pharmacophore-based screening experiments to identify inhibitors of the potassium channel subfamily K member 18 (KCNK18). KCNK18 is one of the determinant factors of migraine-associated pain. A large number of mutations in the KCNK18 gene exist that are associated with excessive neuronal excitability and severe headaches (Grangeon et al., 2023). Sehgal et al. (2014) used pharmacophore models created based on other anti-migraine drugs and the LigandScout tool (Wolber and Langer, 2005) to screen the ZINC database and two other custom-made compound libraries for new KCNK18 inhibitors. The top-ranked compounds were subsequently analyzed using docking with the AutoDock software (Trott and Olson, 2009; Eberhardt et al., 2021), and the top four molecules were chosen for further assessment in binding experiments. Based on docking and drug likeness analysis, newly identified compounds (PB-408318540, PB-415019010, PB-414901730, PB-414901692) were proposed as potential drug molecules to target KCNK18, opening up a therapeutic option for the treatment of migraine occurrences.

In another study, Krueger et al. (2009) tested various ligand-based VS procedures to discover new hits for the N-Methyl-D-Aspartate (NMDA) receptor. NMDA receptor is an ion channel that is found in neurons and activated upon binding of glutamate and glycine. Both the hypo- and hyperfunctioning of NMDA is involved in various neurodegenerative diseases, such as schizophrenia, Parkinson's disease, and Alzheimer's disease (Lin and Lane, 2019). The glycine binding site of the NMDA receptor represents a promising strategy for inhibitor design (Parsons, 2001). Thus, after analyzing all commercially available compound libraries, Krueger et al. (2009) obtained ~4.6 million molecules to screen against the NMDA receptor's glycine site. The authors used 2D and 3D descriptors for screening as well as ligand docking with Glide SP (Friesner et al., 2004) and Glide XP (Friesner et al., 2006), pharmacophore-based and QSAR-based models, and 3D shape search strategies. From each method 500 molecules were extracted, and 201 of them proceeded into *in vitro* testing. While most of the newly identified molecules exhibited a low activity in the micromolar range, all the applied methods were able to derive compounds with novel scaffolds and a high percentage of true actives.

Ion channel researchers have also harnessed machine learning techniques during the early stages of analgesic discovery. This includes identifying novel genes and pathways linked to both acute and chronic pain (Chidambaran et al., 2020), as well as predicting inhibitors for the Na_V_1.7 sodium channel, an important target for the treatment of pain. To simplify the prediction of novel multi-target analgesics or drug combinations for pain management, an extensive pain-focused chemogenomics knowledge base has been established. This comprehensive resource incorporates existing analgesics, the 3D structures of pain-related targets, and compounds associated with these target proteins (Kong et al., 2020).

## Virtual drug discovery with deep generative models

Deep learning is not only used for speeding up VS methods, but is also a driver for the field of generative drug design (also referred to as de novo drug design). In generative drug design, novel chemical molecules with desired chemical and biological properties are generated from scratch, aiming to find new bioactive and synthesizable molecules in a time- and cost-efficient manner. Briefly, the essence of a generative model is to learn the distribution of molecules presented in a training set and generate new molecules for one or multiple targets which are different from those in the training set (Zeng et al., 2022). Combined with evolutionary algorithms or reinforcement learning, the properties of the generated molecules can be further optimized to satisfy different design objectives (Tan et al., 2022). Generative drug design is a relatively new field but could offer advantages compared to conventional VS with regard to the time and cost required for navigating the large chemical space. In a remarkable study by Zhavoronkov et al. (2019) deep generative drug design enabled the discovery of novel potent small molecule inhibitors of the discoidin domain receptor 1 (DDR1) with nanomolar inhibitory efficacy in only 21 days.

The representation of molecules employed by the generative model can be in many forms (David et al., 2020). Many methods use the Simplified Molecular Input Line Entry System (SMILES) (Weininger, 1988) to represent molecules as sequence of characters. From the sequences of SMILES characters as input, language processing neural networks such as recurrent neural networks (RNNs) learn to predict one character at a time, based on the proceeding portion of the sequence and a probability distribution. From the learnt probability distribution new SMILES strings can be sampled (Gupta et al., 2018). However, this approach has one or more limitations. The generated SMILES may not represent a chemically feasible structure, and even a single character change in a SMILES code can change the underlying molecular structure significantly. To overcome these limitations, approaches using graph-based (Li et al., 2018; Xia et al., 2019) and 3D molecule representations (Xie et al., 2022) have been developed. In addition to RNNs (Gupta et al., 2018; Segler et al., 2018), other generative design algorithms include variational autoencoder (VAE) (Gómez-Bombarelli et al., 2018), generative adversarial network (GAN) (Abbasi et al., 2022), transformer models (Liu et al., 2023), and generative models combined with reinforcement learning (RL) (Popova et al., 2018; Liu et al., 2021; Govinda Bhisetti, 2022).

Since drug-likeness and synthetic accessibility are critical parameters that decide about the success of drug candidates, generative models have been trained to yield molecules with specific properties. For example, RL with policy gradient for forward synthesis (PGFS) was proposed as a method to generate molecules that can be feasibly synthesized (Krishna Gottipati et al., 2020). Furthermore, RationaleRL is a graph-based RL model that tries to optimize a multi-objective target function, including properties such as bioactivity against multiple proteins, drug-likeness, and synthetic accessibility (Jin et al., 2020).

The application of deep generative drug design to ion channels is still in its infancy. Schultz et al. (2021) reported the use of deep generative models to design novel antagonists targeting the phencyclidine (PCP) site of the N-methyl D-aspartate receptor (NMDAR). NMDAR antagonists have demonstrated therapeutic benefit in the treatment of neurological diseases such as Parkinson's and Alzheimer's disease (Liu et al., 2019). The authors applied a VAE-based method, called DarkChem (Colby et al., 2020), for NMDAR antagonist design and developed a library of potential NMDAR PCP site-targeting molecules. From ~200,000 compounds designed by DarkChem, 12 novel compounds were found that passed all subsequent *in silico* filtering techniques, including ligand docking, ADMET and synthesizability predictions, drug-likeness filter, substructure and similarity analyses, and were not available in existing public chemical databases. This study provided an example of what generative drug design on ion channels can achieve, although chemical synthesis and experimental validation of the AI-generated compounds were not performed.

**Figure 5:**
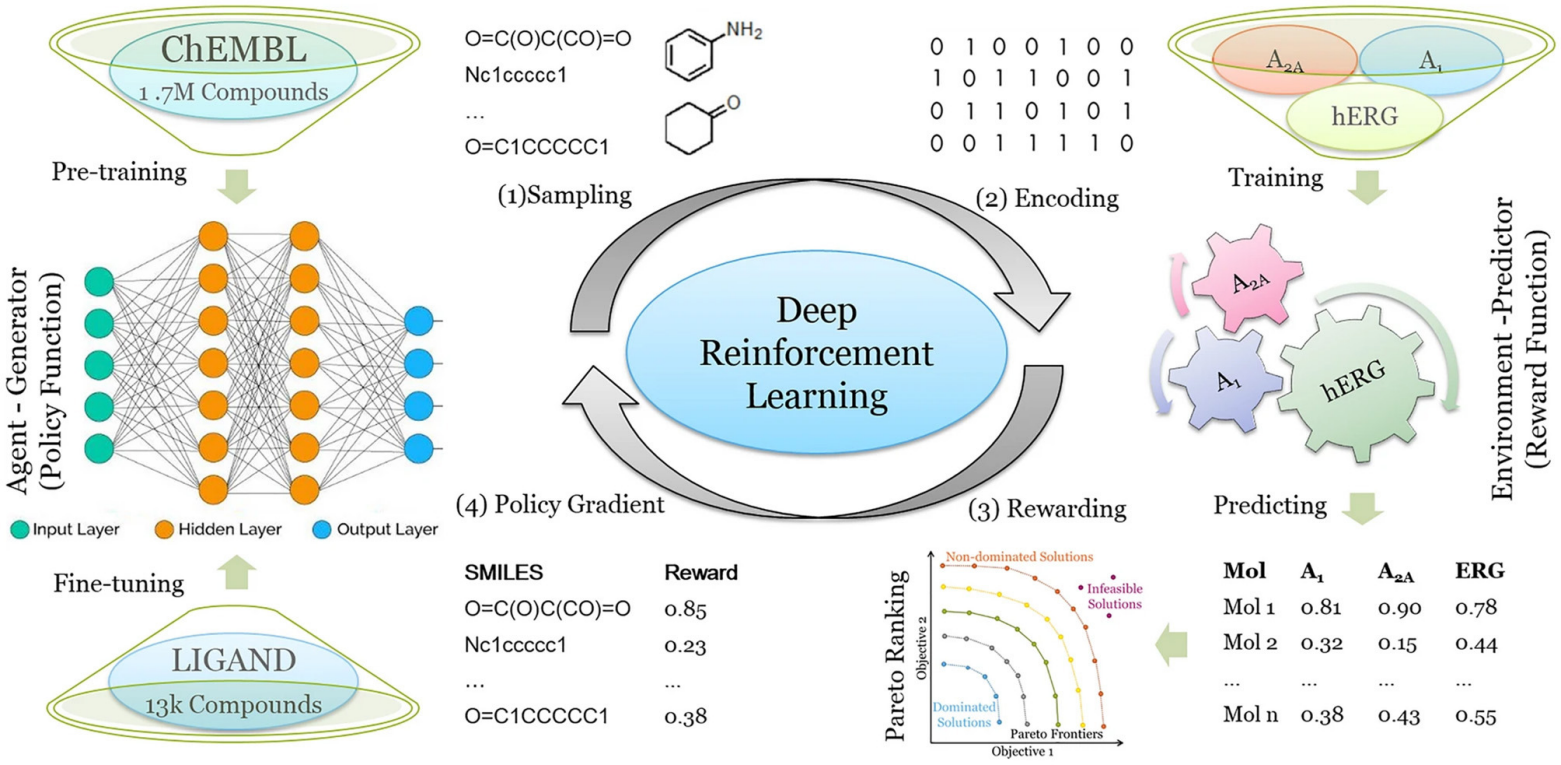
Workflow of the DrugEx method for designing molecules with selectivity to A_1_ and A_2A_ adenosine receptors but no affinity for hERG. (1) New molecules are sampled as SMILES based on the probability calculated by the RNN-based agent generator. (2) The SMILES are encoded into descriptors and their affinity for A_1_, A_2A_ and hERG is predicted. (3) The predicted affinities are transformed into a single value as the reward for each molecule based on Pareto optimization. (4) For training the generative model, the SMILES and their rewards are sent back to the generator. Steps (1) to (4) are repeated until convergence of the training process is reached. Reproduced from Liu et al. (2021) under permission of Creative Commons Attribution 4.0 International License (http://creativecommons.org/licenses/by/4.0/).

To better meet the requirements of drug discovery, deep drug design models are able to consider multiple design objectives. Liu et al. (2021) demonstrated a RNN- and RL-based algorithm, DrugEx, which achieves multi-objective molecule optimization to generate molecules which are active toward one or multiple specific targets while avoiding off-target effects with other proteins. DrugEx was tested for the generation of molecules that should have high affinity for adenosine receptor subtypes A_1_ and A_2A_ but low or zero affinity for the hERG potassium ion channel. Because drug-induced blockage of the hERG ion channel can lead to severe cardiotoxicity, which has been one of the most common reasons for the withdrawal of drugs from the market, hERG toxicity assessment methods have been implemented in the early stages of drug discovery. In the conceptualization of DrugEx, the authors used an RNN as the agent and several machine learning prediction models as environment which operate together in the RL framework. The reward of each molecule is calculated from the Pareto ranking obtained by considering the ML scores for all objectives in the environment as well as a metric representing molecule chemical diversity (Figure 5). The molecules generated by DrugEx covered a larger chemical space compared to other drug design methods and bore some similarity to known adenosine receptor ligands. The approach can be relevant also for developing more selective ion channel modulators.

Molecule design with deep generative models has brought new momentum for drug discovery. If constantly improved and further developed, these methods may be increasingly used for ion channel drug discovery. However, current bottlenecks of AI technologies, such as lack of availability of high-quality data and limited interpretability of the model, currently restrict their application and affect their performance.

## Conclusion and perspective

The integration of computational methods such as VS and deep learning holds great promise in revolutionizing the landscape of drug discovery. These methods, by augmenting traditional experimental high-throughput screening (HTS) techniques, offer a multifaceted approach to drug development. VS techniques, including hit expansion, scaffold hopping, and exploration of uncharted chemical space, demonstrate the potential to uncover novel lead compounds that might have been overlooked within conventional screening libraries. Additionally, the predictive power of computational methods in estimating essential pharmacokinetic and toxicological properties facilitates early identification of promising candidates, significantly streamlining the drug discovery pipeline.

While computational methods offer remarkable insights, it is crucial to acknowledge their synergy with experimental HTS methods. The amalgamation of computational and physical screening tests enriches our understanding of compound behavior in complex biological systems. Physical tests provide indispensable data, especially in relevant biological contexts, aiding in the assessment of properties such as absorption, distribution, metabolism, excretion, and toxicity (ADMET), which remain challenging to simulate accurately computationally.

The continuous evolution of computational techniques prompts us to consider the future of drug discovery. While computational simulations are powerful tools offering valuable insights, they are not yet poised to entirely replace traditional physical screening tests. Instead, the synergy between computational and experimental methods represents the most potent approach. Combining computational and experimental approaches in an iterative and integrative manner often leads to the most effective and comprehensive results. As computational methods advance, the question arises: will they eventually supplant or significantly reduce the need for conventional physical screening tests? This intriguing prospect awaits further exploration, marking an exciting chapter in the ongoing narrative of scientific progress in drug discovery.

## Author contributions

KM: Writing—original draft, Writing—review & editing. PP: Writing—original draft, Writing—review & editing. JM: Writing—review & editing. GK: Writing—review & editing.
